# Electron availability in CO_2_, CO and H_2_ mixtures constrains flux distribution, energy management and product formation in *Clostridium ljungdahlii*

**DOI:** 10.1111/1751-7915.13625

**Published:** 2020-07-21

**Authors:** Maria Hermann, Attila Teleki, Sandra Weitz, Alexander Niess, Andreas Freund, Frank R. Bengelsdorf, Ralf Takors

**Affiliations:** ^1^ Institute of Biochemical Engineering University of Stuttgart Allmandring 31 Stuttgart 70569 Germany; ^2^ Institute of Microbiology and Biotechnology Ulm University Albert‐Einstein‐Allee 11 Ulm 89069 Germany

## Abstract

Acetogens such as *Clostridium ljungdahlii* can play a crucial role reducing the human CO_2_ footprint by converting industrial emissions containing CO_2_, CO and H_2_ into valuable products such as organic acids or alcohols. The quantitative understanding of cellular metabolism is a prerequisite to exploit the bacterial endowments and to fine‐tune the cells by applying metabolic engineering tools. Studying the three gas mixtures CO_2_ + H_2_, CO and CO + CO_2_ + H_2_ (syngas) by continuously gassed batch cultivation experiments and applying flux balance analysis, we identified CO as the preferred carbon and electron source for growth and producing alcohols. However, the total yield of moles of carbon (mol‐C) per electrons consumed was almost identical in all setups which underlines electron availability as the main factor influencing product formation. The Wood–Ljungdahl pathway (WLP) showed high flexibility by serving as the key NAD^+^ provider for CO_2_ + H_2,_ whereas this function was strongly compensated by the transhydrogenase‐like Nfn complex when CO was metabolized. Availability of reduced ferredoxin (Fd_red_) can be considered as a key determinant of metabolic control. Oxidation of CO *via* carbon monoxide dehydrogenase (CODH) is the main route of Fd_red_ formation when CO is used as substrate, whereas Fd_red_ is mainly regenerated *via* the methyl branch of WLP and the Nfn complex utilizing CO_2_ + H_2_. Consequently, doubled growth rates, highest ATP formation rates and highest amounts of reduced products (ethanol, 2,3‐butanediol) were observed when CO was the sole carbon and electron source.

## Graphical abstract

Our studies compared the impact of the gas mixtures CO_2_ + H_2_, syngas and CO on the intracellular flux distribution, energy management, product formation and electron efficiency of *C. ljungdahlii*. The advantage of CO consumption for producing reduced products such as alcohols is pinpointed. In addition, we demonstrated that the Wood‐Ljungdahl pathway adapts its main function depending on the gas mixture consumed.

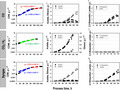

## Introduction

Climate protection and sustainability are two of the greatest challenges for today’s society and for decision‐makers in politics and in industry. In this context, bacterial synthesis gas (syngas) fermentation represents a promising technology for the sustainable production of commodity chemicals and biofuels. It offers a possibility to replace fossil‐based resources while reducing the greenhouse gas carbon dioxide (CO_2_) and utilizing the waste gas carbon monoxide (CO) (Bengelsdorf and Dürre, 2017; Takors *et al*., 2018). For instance, this was already achieved by using exhaust gas of the steel industry as a carbon source (LanzaTech, 2018). Alternatively, syngas, a mixture mainly comprising CO, CO_2_ and hydrogen (H_2_), may be utilized as an inexpensive feedstock because it is derived from agricultural, industrial and municipal wastes (Bengelsdorf and Dürre, 2017; Takors *et al*., 2018). Syngas could be metabolized *via* hydrogenesis, methanogenesis or acetogenesis by a multitude of anaerobic bacteria thereby accessing a wide range of products (Latif *et al*., 2014; Diender *et al*., 2015; Takors *et al*., 2018). One popular representative of gas‐fermenting acetogenic bacteria is *C. ljungdahlii*. The strain was isolated in 1993 from chicken yard waste (Tanner *et al*., 1993). Characteristically, this bacterium shows a wide substrate spectrum. Beside different sugars, it can metabolize syngas autotrophically, CO solely and a mixture of CO_2_ and H_2_ to form the natural products acetate, ethanol, 2,3‐butanediol and lactate (Tanner *et al*., 1993; Köpke *et al*., 2010; Köpke *et al*., 2011). In addition, *C. ljungdahlii* is genetically accessible enabling metabolic engineering for the optimized formation of natural and non‐natural products (Molitor *et al*., 2017; Woolston *et al*., 2018; Huang *et al*., 2019).

As a prerequisite of further strain application, it is crucial to understand energy and redox management in acetogens, as they are known to live at the thermodynamic edge of life (Schuchmann and Müller, 2014). *C. ljungdahlii* uses the WLP for the fixation of CO_2_ and CO (Köpke *et al*., 2010). The pathway consists of two branches and is characterized by the stepwise reduction of CO_2_ to a methyl group that is combined with CO and CoA to generate acetyl‐CoA, consuming one ATP. The conversion of acetyl‐CoA to acetate provides *C. ljungdahlii* one possibility to gain one ATP *via* substrate‐level phosphorylation (Drake et al., 2008; Ragsdale, 2008; Schuchmann and Müller, 2014). Consequently, no net ATP is produced *via* the central metabolism which is why *C. ljungdahlii* relies on a proton gradient coupled to an H^+^‐translocating ATPase to obtain additional ATP. To establish the proton gradient, the membrane ferredoxin:NAD oxidoreductase (Rnf complex) catalyses the oxidation of Fd_red_ while transferring electrons to NAD^+^. For providing crucial reducing equivalents, either oxidation of CO *via* CODH or of H_2_ by a bifurcating hydrogenase (HYD) is used (Müller *et al*., 2008; Köpke *et al*., 2010; Tremblay *et al*., 2012; Wang *et al*., 2013). Reducing equivalents are not only needed to enhance ATP formation, they are electron donors for native products such as ethanol and 2,3‐butanediol, too (Köpke *et al*., 2010; Köpke *et al*., 2011). Therefore, *C. ljungdahlii* can regulate its ATP availability by redirecting the reducing equivalents in different by‐products (Mock *et al*., 2015; Richter *et al*., 2016; Molitor *et al*., 2017; Valgepea *et al*., 2017; Norman *et al*., 2019). Apparently, this links cellular energy formation, growth and by‐product formation with the electron availability in the gas mixture, the core topic of this study. We investigated growth kinetics and by‐product formation of *C. ljungdahlii* as a function of varying substrate compositions. Hence, bioreactor cultivations were performed installing controlled cultivation conditions in 2 l scale using the three different substrates CO, a mixture of CO_2_ and H_2_ and syngas. Intracellular carbon fluxes, redox metabolism and energy management were further characterized using flux balance analysis by coupling the experimentally observed uptake and production rates with a manually reconstructed genome‐scale metabolic model.

## Results

### Comparative autotrophic batch cultivation of *C. ljungdahlii* with different substrate conditions

#### Growth kinetics

**Fig. 1:**
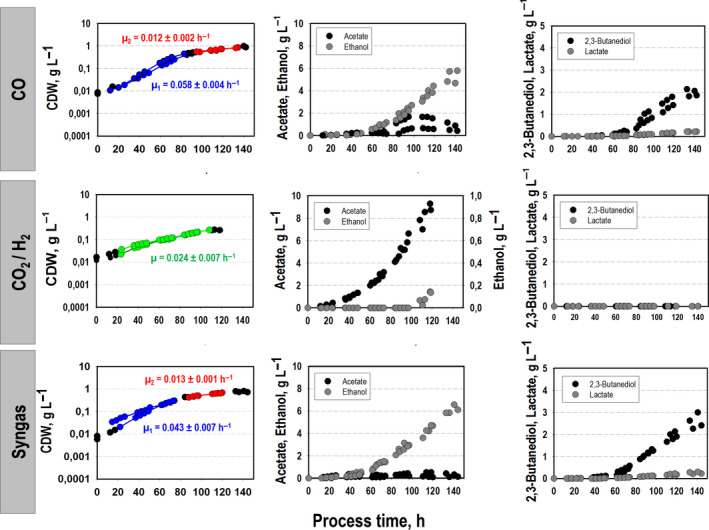
Comparative analysis of growth and product formation of *C. ljungdahlii* based on the conversion of CO, CO_2_ + H_2_ or syngas in steadily gassed batch cultivations using stirred‐tank bioreactor. For each substrate condition, two independent experiments were performed that are demonstrated by individual graphs (*T* = 37 °C; pH = 5.9; *V*_R_ = 3 l; 500 rpm). To determine the respective growth rate, we performed regression fitting for each data set. Growth rates *µ* and coefficients of determination *R* ^2^ are given in the Supplementary Material (Table S1).

**Fig. 2:**
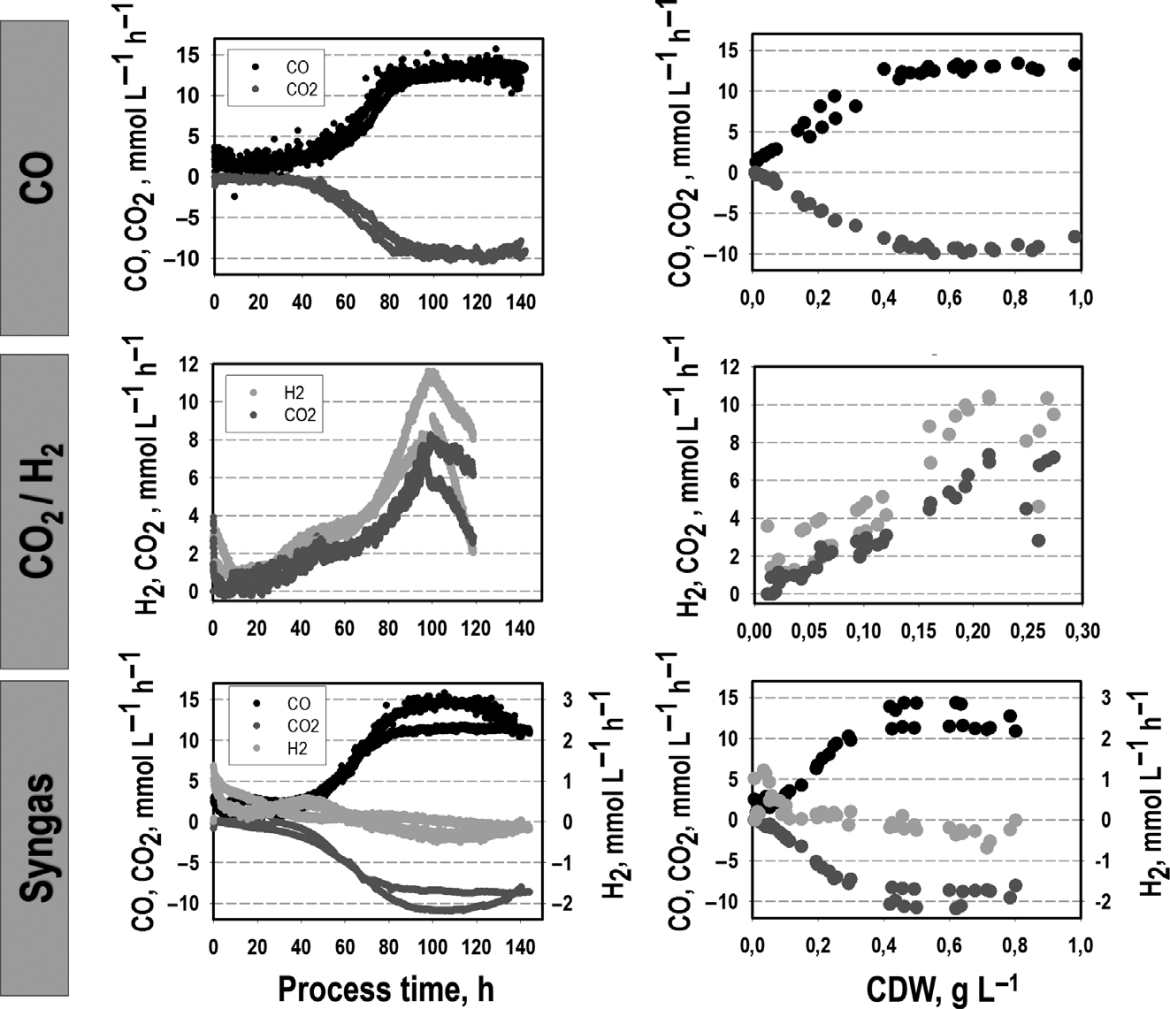
Comparative analysis of the gas uptake of *C. ljungdahlii* based on the conversion of CO, CO_2_ + H_2_ or syngas in steadily gassed batch cultivations using stirred‐tank bioreactor. For each substrate condition, two independent experiments were performed that are demonstrated by individual graphs plotted against the process time and the respective CDW (*T* = 37 °C; pH = 5.9; *V*_R_ = 3 l; 500 rpm).

To gain a better understanding of the carbon and energy metabolism of *C. ljungdahlii,* we compared growth, product formation and substrate uptake for different substrates (Figs 1 and 2). The respective total substrate‐to‐biomass and substrate‐to‐product yields are summarized in Table 2. Batch cultivations were performed in steadily gassed 2 l bioreactors using the compositions **CO**,** CO_2_** **+ H_2_** and **syngas** in duplicates. Detailed gas compositions are described in the Experimental procedures section. In the presence of CO, two growth phases occurred with an exponential growth rate of *µ*_exp_ = 0.06 ± 0.004 h^−1^ [average ± standard deviation] on CO and *µ*_exp_ = 0.04 ± 0.007 h^−1^ on syngas during the first and lowered growth with *µ*_exp_ = 0.01 ± 0.002 h^−1^ on CO and *µ*_exp_ = 0.01 ± 0.001 h^−1^ on syngas during the following period. On contrary, *C. ljungdahlii* showed steady exponential growth with *µ*_exp _= 0.024 ± 0.003 h^−1^ utilizing CO_2_ + H_2_. After approximately 140 h, the maximum cell dry weight (CDW) of 0.93 ± 0.08 g l^−1^ and CDW = 0.76 ± 0.06 g l^−1^ was achieved representing 6.8 ± 0.7% and 2.7 ± 0.1% of totally captured CO and syngas respectively. The CO_2_ + H_2_ approach fixed 3.1 ± 0.1% of captured carbon as biomass reaching maximum CDW = 0.26 ± 0.001 g l^−1^ already after 120 h. In agreement with the growth phenotype, two phases of gas uptake were observed when CO was present. Proportional to the growth, the preferred substrate CO was consumed exponentially yielding a maximum volumetric uptake rate of 12.7 ± 0.4 mmol (l h)^−1^, i.e. *q*_CO_ = 23.8 ± 1.3 mmol (g h)^−1^, both in the CO and the syngas approach. CO uptake was accompanied by a proportional CO_2_ formation achieving a maximum formation rate of 9.6 ± 0.5 mmol (l h)^−1^, i.e. q_CO2_ = 17.9 ± 0.4 mmol (g h)^−1^. In the following growth phase, both volumetric rates remained almost constant. The values are in good accordance with previously described CO uptake and CO_2_ production rates of *Clostridium autoethanogenum* and *C. ljungdahlii* cultivated on syngas in a chemostat mode with a dilution rate of 0.04 h^−1^. The authors determined CO uptake rates of 18.4–30 mmol (g h)^−1^ and CO_2_ formation rates of 4–20 mmol (g h)^−1^ with different biomass concentrations and H_2_ proportions in the substrate gas cultivating *C. autoethanogenum*. For *C. ljungdahlii,* CO uptake rates of 16.6–32.7 mmol (l h)^−1^ and CO_2_ production rates of 2.1–16.6 mmol (l h)^−1^ were reported (Richter *et al*., 2013; Martin *et al*., 2016; Valgepea *et al*., 2017; Valgepea *et al*., 2018). Remarkably, only a low mean H_2_ uptake of 0.29 ± 0.05 mmol (l h)^−1^ was observed in our syngas studies during the first growth period which even turned into H_2_ formation of about 0.30 ± 0.06 mmol (l h)^−1^ in the subsequent phase, whereas a simultaneous utilization of H_2_ and CO is shown in several previous studies describing continuous cultivations performed in a chemostat mode (Richter *et al*., 2013; Martin *et al*., 2016; Valgepea *et al*., 2017; Valgepea *et al*., 2018). In CO_2_ + H_2_ mixtures, both substrates were taken up simultaneously during the exponential growth with the maximum H_2_ uptake rate of 10.35 ± 0.10 mmol (l h)^−1^, i.e. *q*_H2_ = 50.8 ± 0.1 mmol (g h)^−1^ and the maximum CO_2_ uptake rate of 7.16 ± 0.27 mmol (l h)^−1^, i.e. *q*_CO2_ = 35.0 ± 1.2 mmol (g h)^−1^ after about 100 h. Subsequently, a decrease of both uptake rates was observed.

#### Product formation

Depending on the carbon source, different by‐product formation patterns were observed (Table 1). Utilizing CO, acetate, ethanol, 2,3‐butanediol and lactate were produced. Ethanol formation occurred steadily, whereas acetate was only formed during exponential growth and was consumed afterwards leaving 0.68 ± 0.27 g l^ –1^. 2,3‐butanediol production started in the second growth phase together with lactate. Final 2,3‐butanediol and lactate titres were 1.96 ± 0.14 g l^−1^ and 0.21 ± 0.01 g l^−1^ respectively. Related final substrate‐to‐product conversion yields were 0.020 ± 0.01 C‐mole mole_CO_^−1^ for acetate, 0.205 ± 0.01 C‐mole mole_CO_^−1^ for ethanol, 0.078 ± 0.007 C‐mole mole_CO_^−1^ for 2,3‐butanediol and 0.008 ± 3.5*10^−5^ C‐mole mole_CO_^−1^ lactate.

**Table 1:** Maximal growth rates of *C. ljungdahlii* and final by‐product concentrations using CO, CO_2_ + H_2_ or syngas. 2,3‐butanediol are abbreviated by 2,3‐BD. Gas compositions are described in the Experimental procedures section. Rates reflect exponential growth during batch cultivation in steadily gassed stirred‐tank bioreactor. Values indicated mean of duplicates.

Substrate	*µ*_max_, h^‐1^	c_CDW_, g l^−1^	c_Acetate_, g l^−1^	c_Ethanol_, g l^−1^	c_2,3‐BD_, g l^−1^	C_Lactate_, g l^−1^
CO	0.058 ± 0.004	0.93 ± 0.08	0.68 ± 0.27	5.23 ± 0.79	1.96 ± 0.14	0.21 ± 0.01
CO_2_ + H_2_	0.024 ± 0.003	0.26 ± 0.001	9.02 ± 0.39	0.14 ± 0.01	–	–
Syngas	0.043 ± 0.007	0.76 ± 0.06	0.38 ± 0.08	5.91 ± 0.95	4.45 ± 0.64	0.26 ± 0.06

By contrast, CO_2_ + H_2_ consumption mainly caused the growth‐coupled production of acetate and ethanol, the latter in the aftermath of exponential growth. Maximum titres of acetate and ethanol were observed as 9.02 ± 0.39 and 0.14 ± 0.01 g l^−1^ respectively. Related substrate‐to‐product conversion yields were 0.93 ± 0.001 C‐mole mole_CO2_^−1^ for acetate and 0.02 ± 2.0*10^−5^C‐mole mole_CO2_^−1^ for ethanol. No formation of 2,3‐butanediol and lactate was found. Product formation using syngas resembled the CO phenotype although acetate formation was less pronounced. The final substrate‐specific product yields were 0.007 ± 0.004 C‐mole mole_CO_^−1^ for acetate, 0.24 ± 0.004 C‐mole mole_CO_^−1^ for ethanol, 0.11 ± 0.026 C‐mole mole_CO_^−1^ for 2,3‐butanediol and 0.008 ± 0.001 C‐mole mole_CO_^−1^ for lactate.

The determined total consumption and production yields were applied to formulate cellular reduction balances for each experiment (see Supplementary Material). Estimated free Gibbs reaction energies Δ*G_R_* and electron‐specific total product yields are shown in Table 3. While the total product yields remained similar for each substrate condition with around 0.25 C‐mol normalized to the provided electrons, clearly, a larger proportion of electrons was drained into products utilizing CO and syngas compared to CO_2_ + H_2._ The first fixed 62–60% electrons in products, whereas the latter only achieved 50%. In addition, the highest ΔG_R_ was calculated when CO was consumed, underpinning the advantage of preferring CO compared to CO_2_ + H_2_ during batch cultivations.

**Fig. 3:**
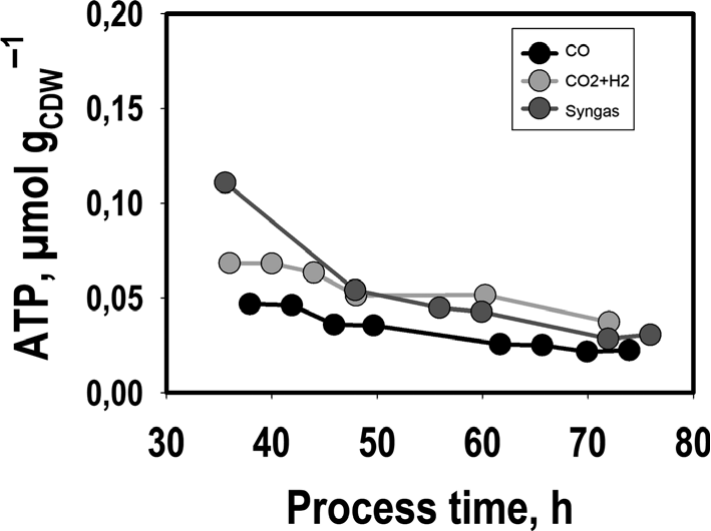
Intracellular ATP levels during exponential growth of *C. ljungdahlii* based on the conversion of CO, CO_2_ + H_2_ or syngas during batch cultivations in steadily gassed stirred‐tank bioreactors (*T* = 37 °C; pH = 5.9; *V*_R_ = 3 l; 500 rpm). ATP levels were determined for one representative experiment of each substrate condition.

Moreover, intracellular ATP pools were measured and depicted in Fig. 3. For each substrate condition, the ATP levels of one independent representative run were determined in order to qualitatively compare the three substrate conditions. All courses showed reducing trends leading to similar mean values of about 0.04 µmol g_CDW_^−1^.

### Simulation of intracellular flux distribution for different substrates

**Fig. 4:**
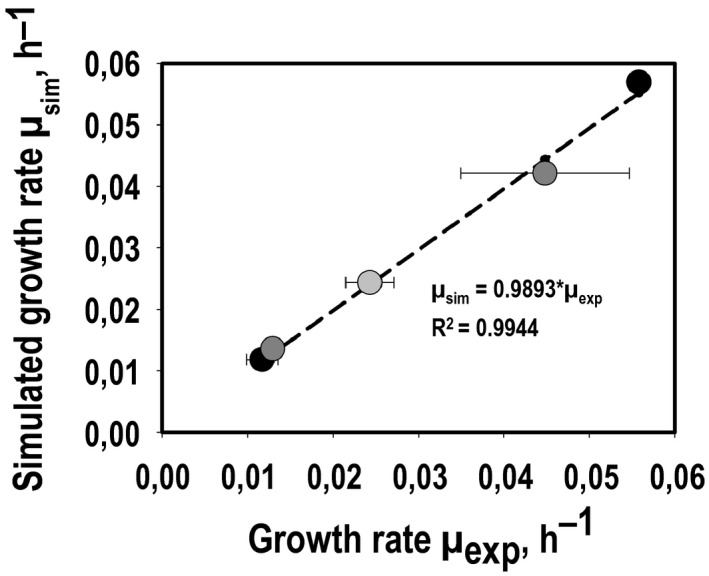
Simulated *versus* measured growth rates of *C. ljungdahlii* converting CO (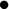), CO_2_ + H_2_ (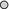) or syngas (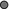) in steadily gassed batch cultivations in stirred‐tank bioreactors. For each substrate condition, two independent experiments were performed (*T* = 37 °C; pH = 5.9; *V*_R_ = 3 l; 500 rpm).

To investigate the links between intracellular carbon distribution, redox metabolism and energy conservation of *C. ljungdahlii,* flux balance analysis was performed. Accordingly, we studied intracellular flux patterns during the ‘pseudo‐steady states’ of the batch cultivations. To restrict the solution space assuring that FBA results reflect real physiological state, we constrained our model with all experimentally determined rates (O’Brien *et al.,* 2015). We have reconstructed a small‐scale stoichiometric metabolic model (rSMM) comprising 117 intracellular and 46 transport reactions thereby balancing 180 metabolites (inner degree of freedom: 12, outer degree of freedom: 22). Mean values of the uptake and secretion fluxes for each substrate condition and growth phase detected (summarized in the Supplementary material, Tables S2 and S3), served as constraints which outlines the importance of accurate measurements. Carbon balances were closed by 103.29 ± 2.67%, 99.97 ± 0.16% and 109.74 ± 2.92% using CO, CO_2_ + H_2_ and syngas respectively. In addition, it was important to validate whether the simulation quality of our modelling approach basically allows a reliable comparison of metabolic fluxes using data of the three different substrate conditions we tested. This quality was checked by plotting simulated growth rates *µ*_sim_ versus experimental observations *µ*_exp_ (Fig. 4) applying growth maximization as objective function. High correlation coefficients R^2^>=0.9944 indicated satisfying quality of the simulated results.

**Fig. 5:**
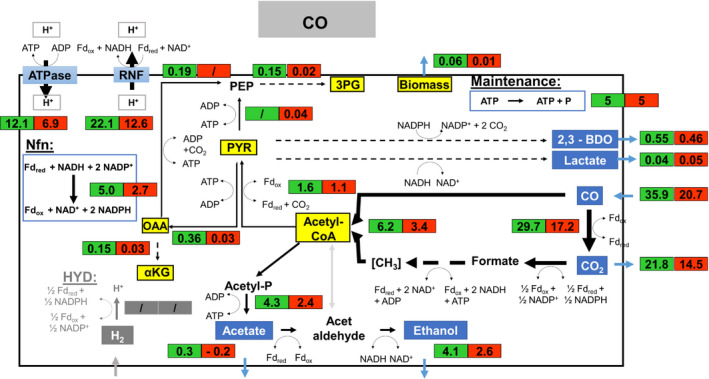
Metabolic flux distributions of *C. ljungdahlii* based on the conversion of CO in steadily gassed batch cultivations in stirred‐tank bioreactors, performed in duplicates. Illustrated are the simulated fluxes in mmol (g_CDW_*h)^−1^ for the first (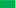) and second (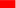) growth phase.

**Fig. 6:**
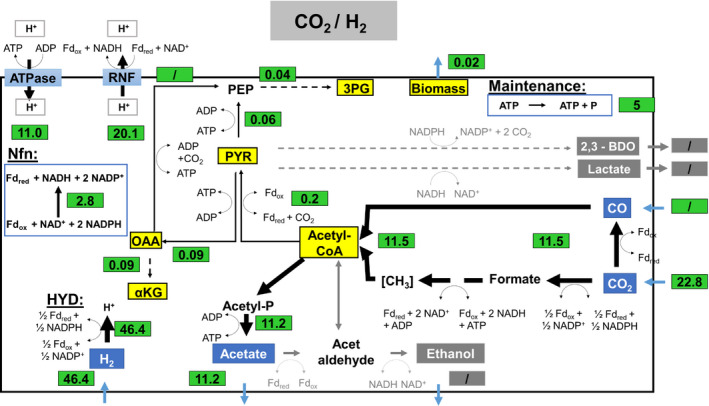
Metabolic flux distributions of *C. ljungdahlii* based on the conversion of CO_2_ + H_2_ in steadily gassed batch cultivations in stirred‐tank bioreactors, performed in duplicates. Illustrated are the simulated fluxes in mmol (g_CDW_*h)^‐1^ for the exponential growth phase.

**Fig. 7:**
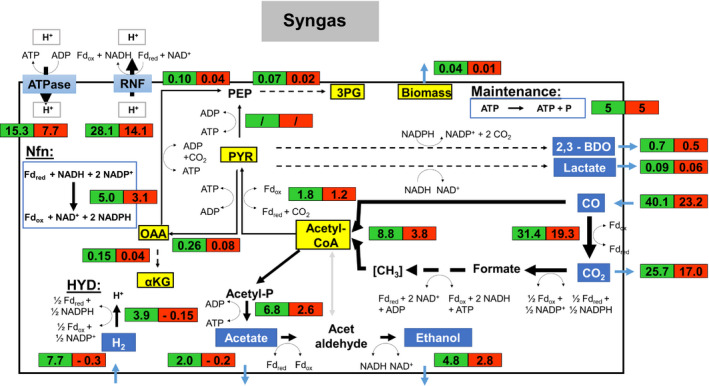
Metabolic flux distributions of *C. ljungdahlii* based on the conversion of syngas in steadily gassed batch cultivations in stirred‐tank bioreactors, performed in duplicates. Illustrated are the simulated fluxes in mmol (g_CDW_*h)^−1^ for the first (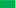) and second (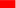) growth phase.

Intracellular metabolic flux distributions of each growth phase are given in Figs 5, 6, 7. To assess the variability of the model calculation, FBA for each cultivation was performed. The results are demonstrated in the Supplementary Material (flux simulations). In addition, a condensed figure showing the fluxes of reducing equivalents and ATP formation normalized to the uptake rate of the respective energy source can be found in the Supplementary Material (Fig. S1). In case of sole CO use, the gas served as carbon and energy (electron) source. Remarkably, about 83% of consumed CO was converted intracellularly to CO_2_ thereby stoichiometrically producing Fd_red_ via CODH. 78% of the produced CO_2_ left the cell which equals 61% of total CO consumed. Accordingly, the function of electron capturing dominated as 83% of CO was capitalized for Fd_red_ formation and only 39% were captured inside serving as carbon source.

The provision of Fd_red_ is the key determinant for the autotrophic metabolism of *C. ljungdhalii,* as it couples ATP synthesis to growth and product formation. First, Fd_red_ is required for reducing NAD^+^ to NADH *via* the Rnf complex and NADP^+^ to NADPH *via* the Nfn complex (Wang *et al*., 2013; Mock *et al*., 2015). In turn, cells need NADH and NADPH to reduce CO_2_ to acetyl‐CoA in the methyl branch of WLP, the latter serving as main precursor for growth and product formation in *C. ljungdahlii* (Köpke *et al*., 2010; Köpke *et al*., 2011). In addition, ATP synthesis is coupled to the Rnf complex. Accordingly, the cellular energy management of *C. ljungdahlii* represents a concerted interplay of Nfn and Rnf complex, the CODH reaction, growth and product formation.

Sixty‐one percent of consumed CO were converted to CO_2_ during the first exponential growth phase. The ratio of the product rates was 0.075: 1: 0.15: 0.01 for acetate, ethanol, 2,3‐butanediol and lactate resulting in the ATP yield of 0.34 mole mole_(CO)_^−1^ and the formation yields of 0.77 mole mole_(CO)_^−1^ for Fd_red_, 0.49 mole mole_(CO)_^−1^ for NAD^+^ and 0.02 mole mole_(CO+H2)_^−1^ for NADPH (Table 4). In the subsequent growth phase, the portion of Fd_red_ formation from CO uptake almost remained similar. In addition, the produced acetate was incorporated again which lead to product formation ratios of 1: 0.2: 0.02 for ethanol, 2,3 butanediol and lactate respectively.

In case of CO_2_ + H_2_ consumption, the formation of Fd_red_ is ensured by the HYD reaction that is accompanied by the regeneration of NADP^+^ to NADPH (Schuchmann and Müller, 2012; Buckel and Thauer, 2013; Wang *et al*., 2013). Additionally, the Nfn reaction is needed to refill the NADP^+^ pool. Remarkably, the Nfn encoded transhydrogenase runs opposite to the CO case, i.e. NADPH is oxidized creating NADP^+^ and NADH. By taking up the substrate gases H_2_ and CO_2_ with a ratio of 2:1, *C. ljungdahlii* can balance the energy and redox metabolism simply by producing acetate. Thereby ATP was formed with the yield of 0.24 mole mole_(H2)_^−1^ and the formation yields of 0.44 mole mole_(H2)_^−1^ for Fd_red_, 0.43 mole mole_(H2)_^−1^ for NAD^+^ and 0.008 mole mole_(H2)_^−1^ for NADPH were achieved.

Utilizing syngas *C. ljungdahlii* makes use of the CODH and the HYD reaction to generate Fd_red_. However, oxidation of H_2_ crucially relies on the availability of NADP^+^. The latter is solely provided by the formate dehydrogenase reaction of the WLP and the 2,3‐butanediol dehydrogenase. In parallel, Nfn and Rnf complexes are used to regenerate the redox metabolites oxidized ferredoxin (Fd_ox_), NADH and NADPH. Growing on syngas 64% of CO is converted to CO_2_ during the first exponential growth phase when the by‐product ratios were 0.4: 1: 0.15: 0.03 for acetate, ethanol, 2,3‐butanediol and lactate respectively. The ATP yield of 0.32 mole mole_(CO+H2)_^−1^ was achieved with the formation yields of 0.72 mole mole_(CO+H2)_^−1^ for Fd_red_, 0.47 mole mole_(CO+H2)_^−1^ for NAD^+^ and 0.014 mole mole_(CO+H2)_^−1^ for NADPH. In the second growth phase, the HYD direction changed. Acetate was taken up, and the proportion of CO converted to CO_2_ increased to 74%. This led to a product formation rate ratio of 1: 0.18: 0.02 for ethanol, 2,3‐butanediol and lactate.

Summarizing, the consumption of CO enabled highest Fd_red_, ATP and NADPH formation yields with respect to the electrons provided (see Table 4). Furthermore, the formation of reduced by‐products was highest, too (see Table 1).

## Discussion

### Growth phenotypes

During autotrophic cultivations, the composition of energy and carbon sources severely influences the growth phenotype and the product spectrum of *C. ljungdahlii*. Our results outline that CO consumption enabled faster growth rates than using gas mixtures of CO_2_ + H_2_. During the first period, growth rates of CO cultures with *µ*_exp_ = 0.06 ± 0.004 h^−1^ more than doubled the rates of CO_2_ + H_2_ with *µ*_exp_ = 0.024 ± 0.003 h^−1^ and were even faster than syngas tests characterized by *µ*_exp_ = 0.04 ± 0.007 h^−1^. The observation reflects that putative CO‐growth inhibition, as outlined by Mohammadi *et al*. (2014), was not active. Apparently, dissolved CO levels were lower than the half‐saturation constant *K*_S_ of 6.33 mmol l^−1^ and the inhibition constant *K*_I_ of 4.44 mmol l^‐1^ identified by Mohammadi *et al*. (2014). Furthermore, CDW values were almost four times higher. The findings are comparable to the observation of Mayer and Weuster‐Botz (2017). They showed that *Clostridium aceticum* yielded almost doubled exponential growth rate and higher CDW concentration using CO instead of CO_2_ + H_2._ By analogy, *C. ljungdahlii* prefers CO because 0.34 mole ATP per mole CO were generated and 7% of captured carbon was used for biomass formation and maintenance needs. On contrary, only 3% of the captured carbon were fixed in biomass and only 0.24 mole ATP per mole H_2_ were gained when utilizing CO_2_ + H_2_. Furthermore, rising acetate concentrations are known to inhibit growth yielding higher cellular maintenance costs by decoupling proton import and ATP synthesis (Jones and Woods, 1986; Valgepea *et al*., 2017). As less ATP is available, utilizing CO_2_ + H_2_ cells are likely to run in ATP shortage (Valgepea *et al*., 2017) finally causing growth collapse. Noteworthy, the final acetate concentration of 9.02 ± 0.39 g l^‐1^ reached with the CO_2_ + H_2_ approach is close to the inhibitory concentration of 12 g l^−1^ postulated by Yang and Tsao (1994) for *Clostridium acetobutylicum*.

### The link between ATP formation and redox balance

The findings anticipate that the consumption of CO led to a higher energy availability than the use of H_2_ as electron source, as already described before (Jack *et al*., 2019). In agreement with this hypothesis, highest ΔG_R_ was calculated when CO was consumed compared to the substrate CO_2_ + H_2_. Interestingly, the intracellular biomass‐specific ATP pools did not differ, indicating that *C. ljungdahlii* is able to balance its energy requirement *via* a tight link between the energy and redox metabolism. A similar characteristic was also postulated for *C. autoethanogenum* before (Marcellin *et al*., 2016). Under balanced growth conditions, formate activation requires ATP which is equilibrated by the formation of acetate. Theoretically, ATP can be further generated *via* oxaloacetate decarboxylation, but this reaction plays only a minor role. Most important, autotrophic ATP production relies on the ATPase activity coupled to the Rnf complex. The respiratory enzyme utilizes Fd_red_ in order to reduce NAD^+^ irrespective of the carbon and electron source used (Müller *et al*., 2008; Tremblay *et al*., 2012). However, the formation of Fd_red_ is dependent on the electron source and differs for CO and H_2._ In case of CO, CODH is applied predominately, whereas the HYD reaction is the key for generating Fd_red_ from H_2_. In addition, Fd_red_ is needed to reduce NADP^+^ to NADPH. The latter greatly influences growth and the by‐product spectrum, as acetogens crucially need to balance NADPH formation with anabolic consumption (Fuhrer and Sauer, 2009). Accordingly, Fd_red_ may be regarded as the key player providing electrons for proper distribution in ATP and products. Besides, the product spectrum has also an important influence on the efficiency of intracellular ATP availability. With increasing acetate formation during growth, the cellular ATP maintenance costs are likely to increase caused by the decoupling of the proton motive force and ATP synthesis (Jones and Woods, 1986; Valgepea *et al*., 2017). The trend of falling intracellular ATP levels consuming CO_2_ + H_2_ (Fig. 3) may reflect the hypothesis. However, even CO consuming cells show similar ATP decline although they may counteract the inhibitory effect of acetate by producing ethanol. Noteworthy, the latter is chaotropic which may cause additional stress *per se* (Valgepea *et al*., 2017). Schatschneider *et al*. (2018) determined intracellular ATP concentration of 0.095 µM g_CDW_^−1^ in *C. autoethanogenum* grown on CO in continuous culture. The value fits well to ATP levels determined in our study. Nevertheless, intracellular ATP levels for heterotrophically cultivated *Clostridium beijerinckii* and *Clostridium acetobutylicum* are higher by 1–2 orders of magnitude (0.55–5.1 µmol g_CDW_^−1^; Meyer and Papoutsakis, 1989; Liu *et al*., 2016). Apparently, this reflects the way of better ATP supply consuming sugars.

### Fd_red_ formation using different gases

The flux balance analysis revealed that with CO the provision of Fd_red_ was ensured by the CODH reaction leading to the stoichiometric regeneration of NAD^+^ to NADH *via* the Rnf complex. Driven by the H^+^ export *via* the Rnf complex, ATP was formed while importing H^+^ (Tremblay *et al*., 2012). Concomitantly, NADPH was produced *via* the Nfn complex, driven by NADH formed *via* the Rnf complex (Mock *et al*., 2015). These findings reflect well the results summarized for *C. autoethanogenum* by Norman *et al*. (2019). Balancing the regeneration of reducing equivalents *via* the WLP 0.77 mole Fd_red_, 0.49 mole NAD^+^ and 0.02 mole NADPH per mole CO was net provided. In essence, these values represent the reducing power and energy equivalents for serving maintenance needs, cellular growth and product formation.

Using CO_2_ + H_2_ as substrate, Fd_red_ was formed by the HYD reaction that is accompanied by the regeneration of NADP^+^ to NADPH. To refill the high need of NADP^+^, the Nfn complex runs opposite to the CO case by oxidizing NADPH transferring electrons to NAD^+^. The latter was fuelled from the conversion of CO_2_ to acetyl‐CoA *via* the WLP. Summarizing, 0.44 mole Fd_red_, 0.43 mole NAD^+^ and 0.008 mole NADPH per mole H_2_ were net provided balancing the Nfn complex, the HYD reaction and the WLP. Obviously, the use of CO_2_ + H_2_ provides fewer reducing equivalents and ATP than the consumption of CO.

These results show the importance of the methyl branch of WLP strongly depended on substrate and electron needs thereby shifting the main purpose: Consuming CO, the branch mainly served as acetyl‐CoA provider and contributed less to Fd_red_ and NAD^+^ formation. The latter was mainly provided by the Nfn complex. In particular, only 8.7% of Fd_red_ and 56.0% of NAD^+^ were drained from the methyl branch (Table 5). On contrast, when H_2_ + CO_2_ was used, the methyl branch supported Fd_red_ formation by 16.5% and gained more importance as NAD^+^ provider: The total NAD^+^ formation relied on this source which clearly illustrates the dual function of the pathway that adapts to current substrate conditions.

When syngas was used, the CO and CO_2_ + H_2_ scenarios somehow overlapped. The activity of the methyl branch was less than the CO_2_ + H_2_ metabolism but higher compared to the CO‐only case. This was due to the combined provision of Fd_red_ by HYD and CODH yielding more reducing power for the reduction of CO_2_. Increasing fluxes through WLP with increased H_2_ supply were also observed by Valgepea *et al*. (2018) investigating continuously cultivated *C. autoethanogenum* on syngas. They predicted direct reduction of CO_2_ with H_2_ to formate by formate‐H_2_ lyase enabling high methyl branch activity with less redox consumption. However, said reaction is not considered in our model, as the *C. ljungdahlii* genome lacks the annotated gene (Köpke *et al*., 2010). Nevertheless, we performed additional model simulations including a formate‐H_2_ lyase for the three different substrate conditions to further assess our findings. The results are summarized in the Supplementary Material (Comparison rSMM_modified rSMM). The simulations show a partly changed flux pattern for syngas and CO_2_ + H_2_ but they support our hypotheses even more. Including formate‐H_2_ lyase activity, the methyl branch supports the Fd_red_ formation by 39.6% on H_2_ + CO_2_ and by 20.5% on syngas. Besides, similar overlapping flux scenarios were also found for the HYD reaction, the Nfn complex and the Rnf complex. Consequently, the output of redox and energy equivalents was better than utilizing CO_2_ + H_2_ but slightly less than using CO as sole electron and substrate donor.

### Product formation

The product‐per‐electron yield in mol‐C was fairly the same in all studies (about 0.25 C‐moles per electron). The observation outlines the fundamental restriction of acetogens offering a maximum product formation that is limited by electron availability (Table 2). However, the product portfolio differed depending on the carbon and electron source. To illustrate the spectrum, the electron efficiency Y_e,e_ was calculated, i.e. the fraction of ‘free electrons’ found in the product versus consumed electrons in the substrates. Utilizing CO solely, about 62% of substrate electrons were found in the products mirroring that the reduction degree of all products was the highest when using CO. The utilization of syngas still showed the same trend but the changing flux distributions (see above) finally lowered the reduction degree of the products. When the mixture of CO_2_ and H_2_ was applied, Y_e,e_ was found to be the lowest, again mirroring the changed intracellular flux distribution.

Acetate turned out to be the dominating product when CO_2_ + H_2_ mixtures were used. The finding coincided with the complete equilibration of ATP use in the methyl branch *versus* ATP formation *via* acetate formation. The exclusive role of the methyl branch as NAD^+^ provider using H_2_ + CO_2_ finally lead to minimal NADH excess to produce reduced products such as ethanol. Furthermore, the ATP gain was the lowest (Table 3). When CO was applied, the methyl branch activities only halved those of H_2_ + CO_2_ and related ATP use was no longer covered by acetate formation. For compensation, NAD^+^ pushed the Rnf complex activity which finally provided strong ATP formation. Interestingly, the net NADH formation using CO outnumbered the needs of the low activity methyl branch which lead to the production of reduced products such as ethanol or 2,3 butanediol (Table 4). The latter represents the highest 2,3‐butanediol production of a batch process with *C. ljungdahlii* described so far (Köpke *et al*., 2011) and reflects well the hypothesis of Norman *et al*. (2019). They predicted high 2,3‐butandiol production under ample carbon but restricted H_2_ supply.

**Table 2:** Final biomass and product yields using CO, CO_2_ + H_2_ or syngas as substrates. 2,3‐butanediol is abbreviated by 2,3‐BD. Gas compositions are described in the ‘Experimental procedures’ the section. Values indicate mean of duplicates.

Substrate	Y_CDW_, _CO/CO2,_	Y_Acetate_, _CO/CO2,_	Y_Ethanol_, _CO/CO2,_	Y _2,3‐BD_, _CO/CO2,_	Y_Lactate_,_CO/CO2,_	Y_CO2_,_CO/CO2,_
	C‐mole mole^−1^	C‐mole mole^−1^	C‐mole mole^−1^	C‐mole mole^−1^	C‐mole mole^−1^	C‐mole mole^−1^
CO	0.068 ± 0.007	0.020 ± 0.01	0.205 ± 0.026	0.078 ± 0.007	0.008 ± 3.5*10^−5^	0.667 ± 0.050
CO_2_ + H_2_	0.031 ± 0.001	0.93 ± 0.001	0.019 ± 2.0*10^−5^	–	–	–
Syngas	0.027 ± 0.001	0.007 ± 0.004	0.24 ± 0.004	0.11 ± 0.026	0.008 ± 0.001	0.714 ± 0.003

**Table 3:** Carbon and electron balances, Gibb’s free reaction energies and electron‐specific overall product yields during growth of *C. ljungdahlii* using CO, CO_2_ + H_2_ or syngas. For gas compositions, see the Experimental procedures section. Rates reflect exponential growth during batch cultivation in steadily gassed stirred‐tank bioreactor. Values indicated mean of duplicates.

Substrate	C ‐ Balance, %	e ‐ Balance, %	Y_Products, e_^−^,	Y_e_^‐^_(Products), e_^−^,	ΔG_R_, kJ
			C‐mole mole^−1^	mole mole^−1^	C‐mole^−1^
CO	103.29 ± 2.67	104.54 ± 3.77	0.24 ± 0.03	0.62 ± 0.02	−47.57 ± 1.17
CO_2_ + H_2_	99.97 ± 0.16	106.33 ± 0.30	0.27 ± 0.002	0.53 ± 0.001	−32.30 ± 3.33
Syngas	109.74 ± 2.92	101.62 ± 7.16	0.24 ± 0.02	0.60 ± 0.04	−38.86 ± 8.70

**Table 4:** ATP, Fd_red_ and NAD^+^ yields derived from flux balance analysis considering exponentially growing *C. ljungdahlii* using CO, CO_2_ + H_2_ or syngas as substrates. The illustrated ATP yields refer to the ATPase activity coupled to the Rnf complex.

Substrate	ATP, mole mole_(CO+H2)_^ −1^	Fd_red_, mole mole_(CO+H2)_^ −1^	NAD^+^, mole mole_(CO+H2)_^ −1^	NADPH, mole mole_(CO+H2)_^ −1^
CO	0.34	0.77	0.49	0.02
CO_2_ + H_2_	0.24	0.44	0.43	0.008
Syngas	0.32	0.72	0.47	0.014

**Table 5:** Percentage provision of NAD^+^ and Fd_red_ and consumption of NADH and NADPH in the methyl branch derived from flux balance analysis considering exponentially growing cells of *C. ljungdahlii* using CO, CO_2_ + H_2_ or syngas as substrates.

Substrate	NAD^+^, %	Fd_red_, %	NADH, %	NADPH, %
CO	56.10	8.66	56.10	92.16
CO_2_/H_2_	100	16.54	100	74.16
Syngas	62.70	9.95	62.70	95.09

Summarizing, our findings confirm the hypothesis that acetogens can regulate their metabolism by coupling the redox metabolism with ATP synthesis and carbon distribution finally yielding different products (Richter *et al*., 2016; Valgepea *et al*., 2017). In this context, we identified the supply and availability of Fd_red_ as the key determinant of metabolic control of *C. ljungdahlii*.

## Conclusion

The metabolism of *C. ljungdahlii* showed high flexibility and adaption for optimum use of CO, CO_2_ + H_2_ and syngas. Clearly, the application of CO as substrate and electron donor is favoured with respect to growth rate, ATP supply and the formation of reduced products. The yield of electrons bound in products compared to electrons used in substrates is the highest for CO. This mirrors the dual role of the methyl branch of the WLP which showed comparably low activity under CO consumption thereby providing excess NADH to produce alcohols. Thereof, the conclusion could be drawn that CO should be the preferred substrate for the production of natural or even non‐natural alcohols with *C. ljungdahlii*. Accordingly, CO‐rich off‐gases of the steel industry should be ideal substrates for the production of alcohols (Sun *et al*., 2019).

## Experimental procedures

### Bacterial strain, growth medium and preculture preparation

*C. ljungdahlii* (DSM 13528) was obtained from the German Collection of Microorganisms and Cell Culture (DSMZ). All experiments were performed under strict anaerobic conditions (Hungate, 1969) using the Tanner mod. PETC medium (ATCC medium 1754) with 15 g l^−1^ MES buffer and 0.5 g l^−1^ yeast extract (Tanner *et al*., 1993). The redox indicator resazurin was solely used for the preculture steps prior to the controlled cultivation in the bioreactor. The preculture seed train contained heterotrophic as well as autotrophic cultivation steps. A frozen cell stock was used to inoculate 5 ml of a heterotrophic medium with 10 g l^−1^ fructose in an unshaken 10 ml anaerobic hungate tube (Glasgerätebau Ochs, Bovenden/Lenglern, Germany) which was incubated for 48 h at 37 °C. Next, 4% (vv^−1^) cell suspension of the preculture was transferred into 50 ml of a heterotrophic medium with 10 g l^−1^ fructose into an anaerobic cultivation bottle with total a volume of 100 ml (Duran protect, Duran group GmbH, Mainz, Germany). The culture was incubated for 48 h at 37 °C and 100 rpm on an orbital shaker (New Brunswick Scientific, Connecticut, USA). In order to adapt the cells to the fructose‐free medium in the bioreactor, an additional preculture step was performed comprising anaerobic cultivation in 500 ml bottles filled with 100 ml fructose‐free medium. Before inoculating the medium with 4 ml preculture, the headspace of the bottles was exchanged three times with the filter‐sterilized gas mixture. Then, 2 bar overpressure was installed and the culture was incubated at 37 °C and 100 rpm. Exponentially growing cells of this seed step were used to inoculate the bioreactor.

### Batch cultivation studies in a stirred‐tank reactor with different substrates

Anaerobic autotrophic batch cultivations with different substrate gas compositions were performed in a fully controlled 3 l stirred‐tank bioreactor (Bioengineering, Wald, Switzerland) with an operational volume of 1.5 l. The bioreactor was equipped with sensors for pH (Mettler Toledo, Columbus, OH, USA), redox potential (Mettler Toledo, Columbus, OH, USA), pressure (Keller AG für Druckmesstechnik, Winterthur, Switzerland) and temperature. LabVieW was used to track process parameters. Temperature was set at 37 °C, and pH was kept constant at 5.9 using 2 M NaOH for titration. Four mass flow controllers (Bronkhorst High‐Tech B.V., Ruurlo, Netherlands) were used to apply different gas mixtures and flows. In the baffled bioreactor, an L‐tube sparger and a six‐blade Rushton impeller stirring with an agitation speed of 500 rpm ensured gas dispersion. The mass spectrometer (Prima Pro, Thermo Fischer Scientific, Waltham, USA) allowed the online measurement of the reactor off‐gas. To avoid foam formation during the batch processes, the antifoam Struktol 674 (Still und Seilacher) was added manually using sterile single‐use syringes and cannulas *via* a septum (Carl Roth GmbH + Co. KG, Karlsruhe, Germany). Three gas compositions were tested in duplicates: (i) **CO** (39% CO; 4% CO_2_; 57% Ar) with a constant gassing rate of 18.9 l h^−1^, (ii) **CO_2_ + H_2_** (47.5% H_2_; 47.5% CO_2_; 5% Ar) with a constant gassing rate of 13.2 l h^−1^ and (iii) **syngas** (55% CO; 30% H_2_; 5% CO_2_; 10% Ar) with a constant gassing rate of 13.2 l h^−1^. As we only had a limited number of gas flow controllers (two at the beginning of our study and three at the end), we partly had to work with predefined gas mixtures. Therefore, it was not possible to establish identical gassing rates and partial pressures of the substrate components for all conditions. We assumed equal supply conditions for all tests, given that average residence time of gas was 11 s in the stirred bioreactor. The latter was estimated considering the reactor diameter and a turbulence factor for the rise velocity of bubbles (Alves *et al*., 2004). To ensure anaerobic conditions, the medium containing bioreactor was sparged with nitrogen with a gassing rate of 60 l h^−1^ applied for 2 h. Off‐gas measurements ensured that oxygen concentrations were always below 0.01% (vv^−1^). Afterwards, the medium was equilibrated with one of the gas compositions (i)–(iii) for 5 h. Two hours prior to inoculation of the bioreactor, sterile reducing agent was added (Tanner *et al*., 1993). During the cultivations, samples were taken frequently to determine the CDW, the extracellular product formation and the intracellular ATP pools.

### Analytical methods

#### Biomass concentration analysis

The optical density was measured offline via a UV/Visible spectrophotometer (Ultrospec 1100 pro, Amersham Bioscience GmbH, Freiburg, Germany) at 600 nm. Thereof, the CDW concentration in [g_CDW_ l^−1^] was correlated with CDW‐to‐OD_600nm_ as 0.25. CDW measurements were based on twice washed (mineralized water) pellets of three 4 ml samples of the cell suspension, centrifuged at 6900 rcf and 4 °C for 5 min (5430 R, Eppendorf, Hamburg, Germany), which were transferred to preweighed glass vials with a total volume of 1.5 ml and dried at 105 °C for at least 24 h in a convection oven (Heraeus, Hanau, Germany). Empty glass vials and the cell pellets were stored in a desiccator (Duran vacuum desiccator, DWK Life Sciences GmbH, Mainz, Germany) for several hours before weighting.

#### Analysis of extracellular products

The detection of the by‐products ethanol, acetate, 2,3‐butanediol and lactate was carried out by an isocratic high‐performance liquid chromatography (HPLC), equipped with a RI detector (1200 Series, Agilent, Santa Clara, CA, USA) and a Rezex ROA‐Organic Acid H^+^ column, at a temperature of 55 °C. A 5 mM H_2_SO_4_ was used as effluent with a flow rate of 0.4 ml min^−1^. Cell‐free samples were generated by centrifugation (5430 R, Eppendorf, Hamburg, Germany) of the cell suspension at 18 000 rcf and 4 °C for 2 min. Subsequently, samples were pretreated consecutively with 4 M NH_3_ and 1.2 M MgSO_4_ solutions to precipitate phosphate and were finally incubated with 0.1 M H_2_SO_4_.

#### Analysis of the intracellular ATP concentrations

Intracellular ATP pool concentrations in [µmol g_CDW_^−1^] were quantified using an HPLC system (1200 Series, Agilent, Santa Clara, CA, USA) coupled to a triple quadrupole tandem mass spectrometer (QQQ‐MS/MS) equipped with an electrospray ion source (Agilent 6410B, Agilent Technologies, Waldbronn, Germany). The method based on a bicratic zwitterionic hydrophilic interaction chromatography (ZIC‐pHILIC) with alkaline (pH 9.2) mobile phase conditions (Teleki *et al*., 2015). The detection was carried out in negative (ESI‐) ionization mode and multiple reaction monitoring (MRM) mode with preoptimized precursor‐to‐product ion transitions (Teleki *et al*., 2015). The ATP standard was obtained from Sigma‐Aldrich (Schnelldorf, Germany). MS‐grade water, methanol, chloroform and acetonitrile were purchased from Carl Roth (Karlsruhe, Germany). Standard stock solutions of ATP were prepared in MS‐grade water and stored at −70 °C. Sample preparation was based on an adapted sequential protocol *via* fast centrifugation treatment (FCT; Plassmeier *et al*., 2007) and a subsequent cold methanol‐chloroform extraction (CME; de Koning and van Dam, 1992). During the exponential growth phase of batch cultures, 5 ml cell suspension was taken periodically as triplicates. The samples were centrifuged at 6900 rcf and −11 °C for 2 min (5430 R, Eppendorf, Hamburg, Germany) and subsequently washed with 5 ml ice‐cold isotonic 0.9% (vv^−1^) sodium chloride solution. Resulting cell pellets and cultivation supernatants (analogues extracellular samples) were immediately quenched by liquid nitrogen and temporarily stored at −70 °C.

Defined cell amounts were resuspended in 200 µl of a precooled 50% (vv^−1^) methanol solution by pulse vortexing (20 s) and merged with 200 µl of precooled chloroform. These extraction solutions were shaken for 1.5 h at −20 °C and subsequently for 1 h at room temperature using a cellmixer (CMV, Labortechnik Fröbel GmbH, Lindau, Germany). After a centrifugation step at 18 000 rcf at −11 °C for 15 min (5430 R, Eppendorf, Hamburg, Germany), the upper aqueous methanol phase (ATP extracts) was removed and temporarily stored at −70 °C.

ATP concentrations were absolutely quantified by a standard‐based external calibration using isotope dilution mass spectrometry (IDMS) as quantification strategy. Prior to analysis, samples and standard mixtures were prepared by constant addition of 10 mM ammonium acetate, 60% (vv^−1^) acetonitrile, 2.5 mM 2‐keto‐3‐deoxy‐6‐phosphogluconate (KDPG) and 8% (vv^−1^) uniformly labelled (U‐13C) *Corynebacterium glutamicum* metabolite extracts (Feith *et al*., 2019). The external calibration range (2.5 nM to 40 µM) with 19 levels was adapted based on previous measurements. Calibration curves were prepared by linear regression of ratios of non‐labelled ATP peak areas and U‐13C‐labelled analogues as internal standard against the respective concentration levels.

KDPG was considered for monitoring of instrumental fluctuations but not for normalization of obtained peak areas.

#### Online analysis of the exhaust gas

The exhaust gas was monitored online by mass spectrometry (Prima Pro, Thermo Fischer Scientific, Waltham, USA). The molar CO, CO_2_ and H_2_ concentrations produced or consumed by *C. ljungdahlii* in the course of the different cultivations were determined by taking into account the adjusted inlet flow rates of the individual gases as well as the measured gas concentrations in the inlet and outlet gas streams (formula 1). In each case, Argon was used as inert gas.

(1) $$r_{i}\left(t\right)=\frac{p}{R\cdot T}\cdot \frac{V_{g,in}}{V_{R}}\cdot \left(\frac{c_{i,\;in}}{100}‐\frac{c_{Ar,\;in}}{c_{Ar,\;out}}\cdot \frac{c_{i,\;out}}{100}\right)$$

*R_i _(t)* represents the volumetric uptake rate of the respective gas at one time point of the cultivation process. For the values of the pressure *p*, the temperature *T* and the gas constant *R* normal conditions are assumed. *V_g,inlet_* reflects the aeration rate, and *V_R_* is the working volume of the reactor. To account for dissolved CO_2,_ total inorganic carbon in cell‐free filtrates was measured using a TC analyser (Multi N/C 2100s, Analytik Jena, Jena, Germany) as described in Graf *et al*. (2018). To calculate the molar concentrations of the gases produced or consumed by the cells as function of the process time, the volumetric rates were integrated.

### Determination of cell specific rates

The biomass‐specific substrate uptake and product formation rates were calculated by dividing the exponential growth rate *µ* by the biomass substrate yield *Y_X/S_* or the biomass product yield *Y_X/P_* respectively (formulas 2 and 3). In prior, the exponential growth rate *µ* was determined graphically by a linear fit of the semilogarithmic plot of the calculated CDW as function of the process time. To deduce graphically the biomass substrate yields as well as the biomass product yields, a linear regression of the substrate or product concentration curves as function of the biomass concentration was applied.

(2) $$q_{S}=\frac{\mu }{Y_{\mathrm{XS}}}$$

(3) $$q_{P}=\frac{\mu }{Y_{\mathrm{XP}}}$$

In addition, electron‐specific overall product yields were calculated (formulas 4 and 5) by assuming that one mole CO_2_ produced and/or one mole H_2_ incorporated yield two electrons that can be transferred to reducing equivalents (Müller, 2003).

(4) $$Y_{\text{products},e^{‐},}=\frac{\sum c_{\mathrm{Pi}}}{\left(c_{{\text{CO}}_{2}}+c_{H_{2}}\right)*2}$$

(5) $$Y_{e^{‐}\left(\text{Products}\right),e^{‐},}=\frac{\sum c_{\mathrm{Pi}}*k_{i}^{*}}{\left(c_{{\text{CO}}_{2}}+c_{H_{2}}\right)*2}$$

*C_Pi_* represents the molar concentration of the respective product, *c*_CO2_ and *c*_H2_ are the molar concentrations of CO_2_ produced and H_2_ consumed at the end of the respective cultivation, and κ_i_* reflects the degree of reduction per carbon of the respective product.

### Determination of the Gibbs free energy changes *Δ*G_R_

In order to evaluate the energy yield of the different analysed substrate conditions, the Gibbs free reaction energy change Δ*G_R_* was determined as described by (Villadsen *et al*., 2011; formula 4) and (formula 5).

(4) $$\Delta G_{R}={\left(\mathrm{iTOTO}\sum_{i}Y_{Pi/S}*\Delta G_{c,i}\right)}_{\text{products}}‐{\left(\mathrm{iTOTO}\sum_{i}Y_{Si/S}*\Delta G_{c,i}\right)}_{\text{substrates}}$$

(5) $$\Delta G_{\mathrm{Ci}}=‐94.4\kappa_{i}^{*}+86.6\;\text{kJ}{\left(C‐\text{mole}\right)}^{‐1}$$

Δ*G_ci_* is the free energy of combustion of the reactant *i*, that is dependent on the respective degree of reduction κ_i_*. The experimental coefficients *Y_Pi/S_* and *Y_Si/S_* reflect the yields of products and substrates in mol‐C. The respective coefficients for H_2_O, NH_3_ and H^+^ were estimated assuming elemental balances. The biomass composition was assumed according to Alberty (1998). A detailed description is attached in the Supplementary Material.

### Stoichiometric metabolic modelling

#### Stoichiometric network reconstruction

Based on the genome annotation of *C. ljungdahlii* (Köpke *et al*., 2010), the genome‐scale models of *C. ljungdahlii* (Nagarajan *et al*., 2013) and of the related clostridial strain *C. acetobutylicum* (Senger and Papoutsakis, 2008), we manually reconstructed a reduced stoichiometric metabolic model (rSMM) using the combined information from the databases KEGG and MetaCyc, literature and own experimental results. In addition, we compared our model with the metabolic model of *C. autoethanogenum* (Marcellin *et al*., 2016). The network was reconstructed, checked for consistency and topologically investigated by means of the Insilico Discovery™ platform (Insilico Biotechnology AG, Stuttgart, Germany). For the sake of simplicity, biosynthetic pathways of glycerophospholipids, cell wall, DNA and nucleotides were lumped. To cover maintenance demands, the growth‐associated value (GAM) of 46.7 mmol ATP per g CDW was installed (Nagarajan *et al*., 2013), whereas non‐growth‐associated maintenance (NGAM) was set to 5 mmol (g_CDW_*h)^−1^. This value corresponds to the mean maintenance cost identified for the closely related acetogen *C. autoethanogenum* on CO, syngas and CO_2_ + H_2_ (Valgepea *et al*., 2018; Heffernan *et al*., 2020). In addition, we made the following assumptions: (i) The ATP synthase reaction is characterized by a conservative stoichiometry of 3.66 H^+^ per 1 ATP (Mock *et al*., 2015); (ii) the HYD is an electron‐bifurcating, NADP^+^ and ferredoxin‐dependent enzyme that uses 2 H_2_ for the reversible reduction of NADP^+^ and Fd_ox_ (Mock *et al*., 2015); (iii) the formate dehydrogenase reaction is based on NADPH and Fd_red_ as electron donor (Schuchmann and Müller, 2014); (iv) the membrane‐bound Rnf complex translocates 2 protons *via* the Fd_red_:NAD^+^ oxidoreductase reaction (Perez *et al*., 2013); and (v) the methylene‐H_4_F reductase reaction catalyses the reduction of methylene‐H_4_ presumably coupled to the reduction of ferredoxin Fd_ox_ with NADH (Köpke *et al*., 2010; Mock *et al*., 2015). Accordingly, Fd_ox_, ATP and 2 NADH are converted to Fd_red_, ADP and 2 NAD^+^. The direct reduction of CO_2_ with H_2_ to formate by formate‐H_2_ lyase was not considered in our model, as the *C. ljungdahlii* genome lacks the annotated gene (Köpke *et al*., 2010). However, formate‐H_2_ lyase activity was identified for CO consuming *C. autoethanogenum* (Wang *et al*., 2013). Therefore, an additional model containing the aforementioned reaction (modified rSMM) was used to further assess our simulative results. Both models are attached in the Supplementary Material.

#### Flux balance analysis

Flux balance analysis (FBA) was performed to investigate the intracellular carbon, redox and energy balances of *C. ljungdahlii* as a function of varying substrate conditions (Schilling *et al*., 2000; Orth *et al*., 2010). This was necessary as the degree of freedom of the model exceeds the maximal number of quantifiable fluxes. For simulations, the Insilico Discovery™ platform was used. Maximization of biomass production was set as objective function, while all experimentally determined extracellular substrate uptake and product formation rates were used as constraints. YE was not considered for modelling, as only 0.5 g l^−1^ YE was provided in the batch medium accounting for < 5% of the incorporated carbon during all cultivations we described. This is reflected by well‐closed balances for measured carbon without YE consideration. Total organic carbon content in YE was measured using a TC analyser (Graf *et al*., 2018). We normalized the sum of the product fluxes to the sum of substrate fluxes (set to 100%) and analysed the relative distributions. Due to the reversibility of the phosphotransacetylase, the acetate kinase, aldehyde oxidoreductase and the acetaldehyde/alcohol dehydrogenase reactions, non‐realistic futile cycling may occur biasing ATP formation. To prevent non‐wanted ATP futile cycles *via* acetate and ethanol formation, the following constraint was additionally set: We limited the acetyl‐CoA synthase flux (*r*_ACCOAS_) by the simulated maximum using experimentally observed substrate uptake rates. Consequently, this maximum also limited the phosphotransacetylase reaction (*r*_PTA_) which, again, served as upper limit for the acetate kinase reaction (*r*_ACK_). Accordingly, a two‐step simulation was performed preventing non‐wanted futile cycling.

## Conflict of interest

None declared.

## Supporting information


**Fig. S1.** Metabolic fluxes of reducing equivalents and ATP formation for growth of *C. ljungdahlii* based on the conversion of CO in the first (A) and second growth phase (B), on CO_2_+H_2_ (C) or syngas in the first (D) and second growth phase (E). The simulated rates were normalized to the respective uptake rate of the energy source (CO or H_2_). For each substrate condition two independent steadily gassed batch cultivations in stirred‐tank bioreactors were performed (T = 37°C; pH = 5.9; *V*_R_ = 3 l; v = 500 rpm).

**Table S1.** Determination of the exponential growth rate for each data set and growth phase observed using regression fitting. Summarized are the determined growth rates µ and the corresponding coefficients of determination *R* ^2^ for the cultivation experiments with the three substrate gases CO (39% CO, 4% CO_2_, 57% Ar), CO_2_+H_2_ (47.5% H_2_, 47.5% CO_2_, 5% Ar) and syngas (55% CO, 30% H_2_, 5% CO_2_, 10% Ar) performed in duplicates.
**Table S2.** Experimentally determination of the substrate uptake and product formation rates rate for each data set and growth phase detected. Summarized are the biomass substrate and biomass product yields Y_x/s_ and Y_X/P_, the corresponding coefficients of determination R^2^ as well as the subsequently determined substrate uptake and product formation rates for the cultivation experiments with the three substrate gases CO (39% CO, 4% CO_2_, 57% Ar), CO_2_+H_2_ (47.5% H_2_, 47.5% CO_2_, 5% Ar) and syngas (55% CO, 30% H_2_, 5% CO_2_, 10% Ar) performed in duplicates.
**Table S3.** Mean values of the uptake and secretion fluxes used as constraints for FBA and the corresponding standard deviations for the cultivation experiments with the three substrate gases CO (39% CO, 4% CO_2_, 57% Ar), CO_2_+H_2_ (47.5% H_2_, 47.5% CO_2_, 5% Ar) and syngas (55% CO, 30% H_2_, 5% CO_2_, 10% Ar) performed in duplicates.
