# Supplementary figures and images for: Electron availability in CO2, CO and H2 mixtures constrains flux distribution, energy management and product formation in Clostridium ljungdahlii
Source: Microb Biotechnol. 2020 Jul 21;13(6):1831–46. doi: 10.1111/1751-7915.13625 (PMC7533319; doi:10.1111/1751-7915.13625)

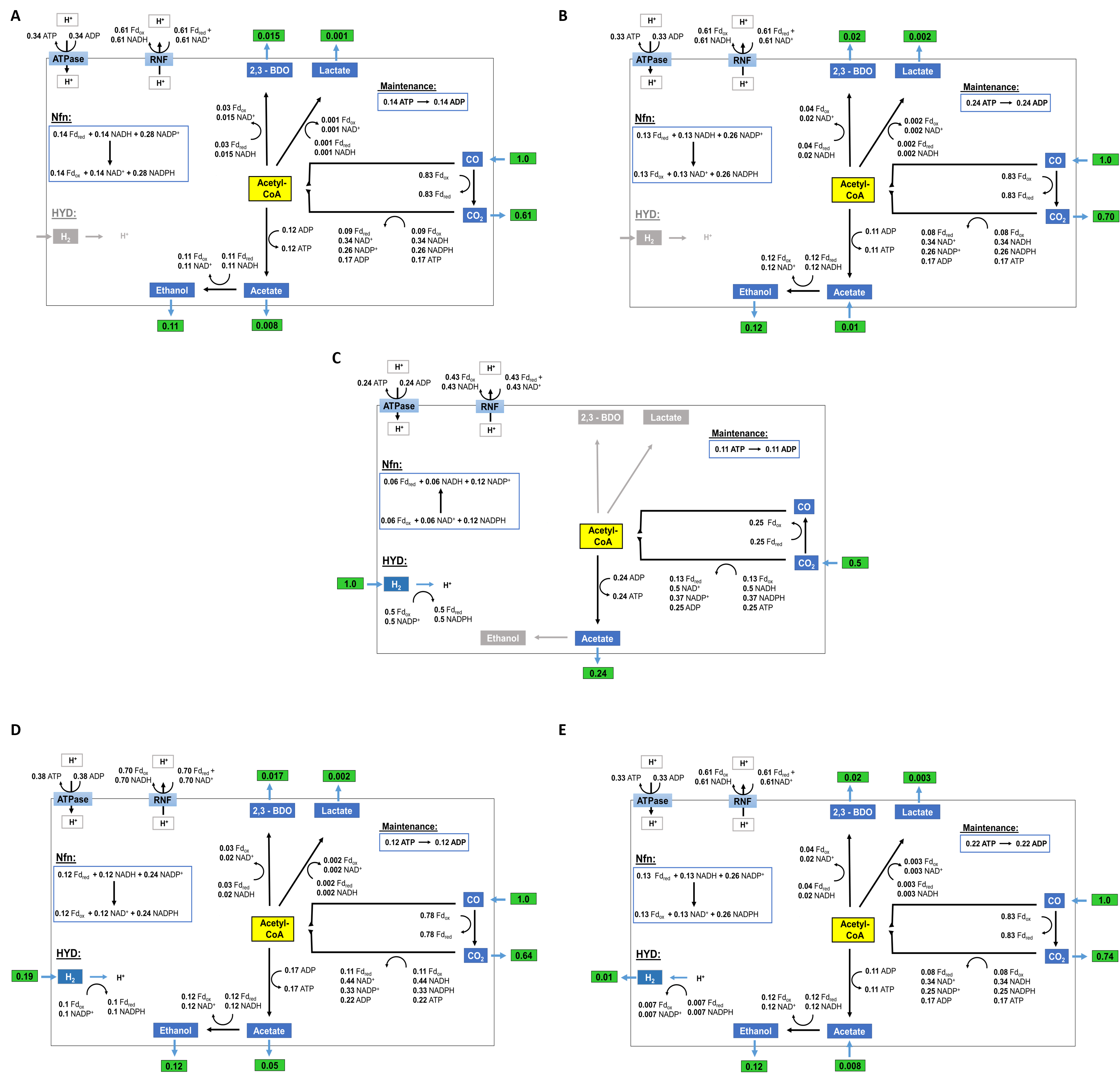

Supplement: Supplementary file 1 — Fig. S1. Metabolic fluxes of reducing equivalents and ATP formation for growth of C. ljungdahlii based on the conversion of CO in the first (A) and second growth phase (B), on CO2+H2 (C) or syngas in the first (D) and second growth phase (E). The simulated rates were normalized to the respective uptake rate of the energy source (CO or H2). For each substrate condition two independent steadily gassed batch cultivations in stirred‐tank bioreactors were performed (T = 37°C; pH = 5.9; V R = 3 l; v = 500 rpm). [file MBT2-13-1831-s001.png]
